# Intra-articular bone marrow mononuclear cell therapy improves lameness from naturally occurring equine osteoarthritis

**DOI:** 10.3389/fvets.2023.1256284

**Published:** 2023-10-09

**Authors:** J. Blake Everett, Bruno C. Menarim, Sarah H. Barrett, Sophie H. Bogers, Christopher R. Byron, R. Scott Pleasant, Stephen R. Werre, Linda A. Dahlgren

**Affiliations:** ^1^Department of Large Animal Clinical Sciences, Virginia-Maryland College of Veterinary Medicine, Virginia Tech, Blacksburg, VA, United States; ^2^Gluck Equine Research Center, Department of Veterinary Science, Martin-Gatton College of Agriculture, Food and Environment, University of Kentucky, Lexington, KY, United States; ^3^Department of Biomedical Sciences and Pathobiology, Virginia-Maryland College of Veterinary Medicine, Virginia Tech, Blacksburg, VA, United States; ^4^Laboratory for Study Design and Statistical Analysis, Virginia-Maryland College of Veterinary Medicine, Virginia Tech, Blacksburg, VA, United States

**Keywords:** BMNC, macrophage, osteoarthritis, synovitis, inflammation

## Abstract

Osteoarthritis (OA) can be debilitating and is related to impaired resolution of synovial inflammation. Current treatments offer temporary relief of clinical signs, but have potentially deleterious side effects. Bone marrow mononuclear cells (BMNC) are a rich source of macrophage progenitors that have the ability to reduce OA symptoms in people and inflammation in experimentally-induced synovitis in horses. The objective of this study was to evaluate the ability of intra-articular BMNC therapy to improve clinical signs of naturally occurring equine OA. Horses presenting with clinical and radiographic evidence of moderate OA in a single joint were randomly assigned to 1 of 3 treatment groups: saline (negative control), triamcinolone (positive control), or BMNC (treatment group). Lameness was evaluated subjectively and objectively, joint circumference measured, and synovial fluid collected for cytology and growth factor/cytokine quantification at 0, 7, and 21 days post-injection. Data were analyzed using General Estimating Equations with significance set at *p* < 0.05. There were no adverse effects noted in any treatment group. There was a significant increase in synovial fluid total nucleated cell count in the BMNC-treated group on day 7 (median 440; range 20–1920 cells/uL) compared to day 0. Mononuclear cells were the predominant cell type across treatments at all time points. Joint circumference decreased significantly in the BMNC-treated group from days 7 to 21 and was significantly lower at day 21 in the BMNC-treated group compared to the saline-treated group. Median objective lameness improved significantly in the BMNC group between days 7 and 21. GM-CSF, IL-1ra, IGF-1, and TNF-α were below detectable limits and IL-6, IL-1β, FGF-2 were detectable in a limited number of synovial fluid samples. Inconsistent and limited differences were detected over time and between treatment groups for synovial fluid PGE_2_, SDF-1, MCP-1 and IL-10. Decreased lameness and joint circumference, coupled with a lack of adverse effects following BMNC treatment, support a larger clinical trial using BMNC therapy to treat OA in horses.

## Introduction

Osteoarthritis (OA) is a common and debilitating disease affecting people and domestic animals (1–4). Among US adults over 15 years of age, arthritis is the most frequent disability (4) and costs more than $300 billion annually in the US (1). OA is the most common cause of lameness and poor performance in horses, accounting for approximately 60% of musculoskeletal problems (2, 5) and affects >80% of dogs over 8 years of age (6) and 90% of cats over 12 years of age (7). Pain and lameness resulting from OA universally cause disability, decreased performance, and interfere with an active lifestyle (2, 4, 5, 7). Treatment for OA is challenging due to limitations in available treatments and an incomplete understanding of disease pathophysiology and how to effectively modify it.

OA is characterized by progressive degeneration of all synovial tissues, including cartilage, synovium and bone (2, 8, 9). However, it is synovial membrane inflammation that is the key driver of the clinical signs and pathologic processes in OA (10–13). Macrophages are the central mediators of synovial inflammation and play a significant role in the development and progression of OA (14–18). However, macrophages are equally important in maintaining joint homeostasis (19, 20). Under physiological conditions, macrophages play a key role in chondrocyte function, phagocytosis, growth factor and cytokine production and inflammation resolution (10, 11, 15, 16, 19–23).

Current therapeutics for OA primarily improve clinical signs by temporarily blocking inflammation, but fail to produce tissue repair or sustained improvement. These treatments are strictly anti-inflammatory, inhibiting both pathological and common homeostatic pathways, and thus can have detrimental side effects (24–26). Corticosteroids, for example, block common cellular pathways (e.g., prostaglandins and NF-kB) innately required for efficient tissue homeostasis, healing and inflammation resolution (24, 27). Inflammation resolution is not only the passive termination of inflammation, but rather a phenomenon that metabolizes pro-inflammatory mediators from acute inflammation, giving origin to pro-resolving lipid mediators that ends leukocyte infiltration, downregulates pro-inflammatory mediators, and clears apoptotic cells and debris. Thus, the indiscriminate use of anti-inflammatory drugs can often impair the establishment of a proper pro-resolving response, leading to chronic inflammation and disease progression (26). Orthobiological therapies, such as autologous conditioned serum and mesenchymal stem cells, have become popular and exert pro-resolving effects, but have shown inconsistent results in clinical studies, with only modest benefits (28–31). There is a critical need for therapies that halt disease progression and resolve synovial inflammation, while maintaining the homeostatic and reparative functions of the joint.

Bone marrow mononuclear cells (BMNC) are a source of macrophage progenitors (>50% naïve cells) to enhance tissue repair and promote inflammation resolution (18, 32–41). BMNC are autologous, can be rapidly isolated from bone marrow aspirate for point-of-care administration, and have the potential to substantially advance the treatment of joint disease (18, 34, 41–44). The pro-resolving effects of bone marrow-derived macrophages have been associated with increased production of interleukin (IL)-10, insulin-like growth factor (IGF)-1 and lipid mediators (41, 44–48). IL-10 is a potent anti-inflammatory cytokine essential in tissue homeostasis, repair and protection of chondrocytes from inflammatory insults (49, 50). Harnessing the pro-resolving properties of BMNC and increasing the number of cells responsible for joint homeostasis may aid in restoration of joint health. BMNC were safely injected into people with knee OA and provided significant decreases in MRI scores for bone marrow edema, cartilage and synovitis and superior improvements in pain, activity level, and quality of life when compared to hyaluronic acid (42, 43). Horses with experimental synovitis had no adverse reactions to intra-articular BMNC and treated joints showed marked gross improvement associated with increasing regulatory macrophages and synovial fluid IL-10 concentrations compared with saline-treated controls (41). BMNC-treated joints were histologically comparable to healthy joints while saline-treated controls remained abnormal (41). Transcriptional and histochemical investigations further revealed that BMNC induce resolution through enhanced expression of homeostatic mechanisms that combine a fine-tuned expression of pro- and anti-inflammatory mediators (40). The objective of this study was to evaluate the ability of intra-articular BMNC therapy to improve clinical signs of naturally occurring osteoarthritis in horses. We hypothesized that intra-articular administration of BMNC would reduce lameness and joint inflammation comparable to that achieved with intra-articular corticosteroids.

## Materials and methods

### Study design

The protocol for this multi-center, randomized, blinded, placebo-controlled clinical trial was approved by the Institutional Animal Care and Use Committee and the Hospital Board. The study was performed at the Virginia-Maryland College of Veterinary Medicine in collaboration with equine practices in the mid-Atlantic region and North Carolina. Adult horses 3–16 years of age referred with lameness isolated to a single joint in one limb were prospectively enrolled. Eligibility for inclusion was based on the following criteria: complete medical record, including signalment, history, and presenting physical examination findings (temperature, pulse, respiratory rate); single limb lameness of Grade 2 or 3 on a scale of 0–5 (Table 1) (51) isolated to a single joint and yielding a positive response (>50% improvement) to intra-articular anesthesia and radiographic evidence of moderate OA. The source of lameness was localized using a combination of subjective lameness evaluation, including limb flexions, and objective quantification using a body-mounted, wireless, inertial sensor system (Lameness Locator^®^), diagnostic intra-articular analgesia, and radiography. Radiographic OA scores (mild, moderate, or severe) were assigned by consensus between 4 experienced clinicians (SHB, CRB, LAD, RSP) (28, 52). Moderate OA was assigned based on radiographic evidence of osteophytes, sclerosis, and lysis, with or without joint narrowing (28, 52). Once selected for study inclusion, a physical examination and complete blood count were performed to ensure general health. Screening of horses for possible study inclusion (physical examination, radiographs, ± intra-articular anesthesia) was performed by our local DVM contacts who helped recruit cases. Final inclusion in the study was determined based on a complete evaluation (physical examination and response to intra-articular anesthesia) was performed by a single investigator (JBE) with assistance from BCM. All initial and all follow-up evaluations were performed by the same single investigator (JBE) with assistance from others (BCM, LAD, SHB). Initial evaluations for study inclusion were performed on-farm. Following the 7-day washout period, horses received the day 0 baseline lameness evaluation and treatment at 1 of 2 locations, the VMCVM or a single veterinary hospital in Pennsylvania. Horses treated in PA were maintained at the PA location for the duration of the study, where all re-evaluations occurred. Horses treated initially at the VMCVM were monitored in-hospital for 48 h prior to being discharged to their home farm with instructions for after-care. Follow-up evaluations were performed at either the home farm or a participating local veterinary hospital.

**Table 1:** Lameness scoring criteria based on observation of the horse at a trot in hand, in a straight line, on a firm or hard surface (51).

Score	Description
0	No lameness observed
1	Mild lameness observed when trotted in straight line (head nod/pelvic hike inconsistent)
2	Obvious lameness observed (head nod/pelvic hike seen consistently)
3	Overt lameness (pronounced head nod/pelvic hike of several centimeters)
4	Severe lameness (extreme head nod/pelvic hike; horse can still be trotted)
5	Horse is non-weight-bearing and should not be trotted

**Figure 1:**
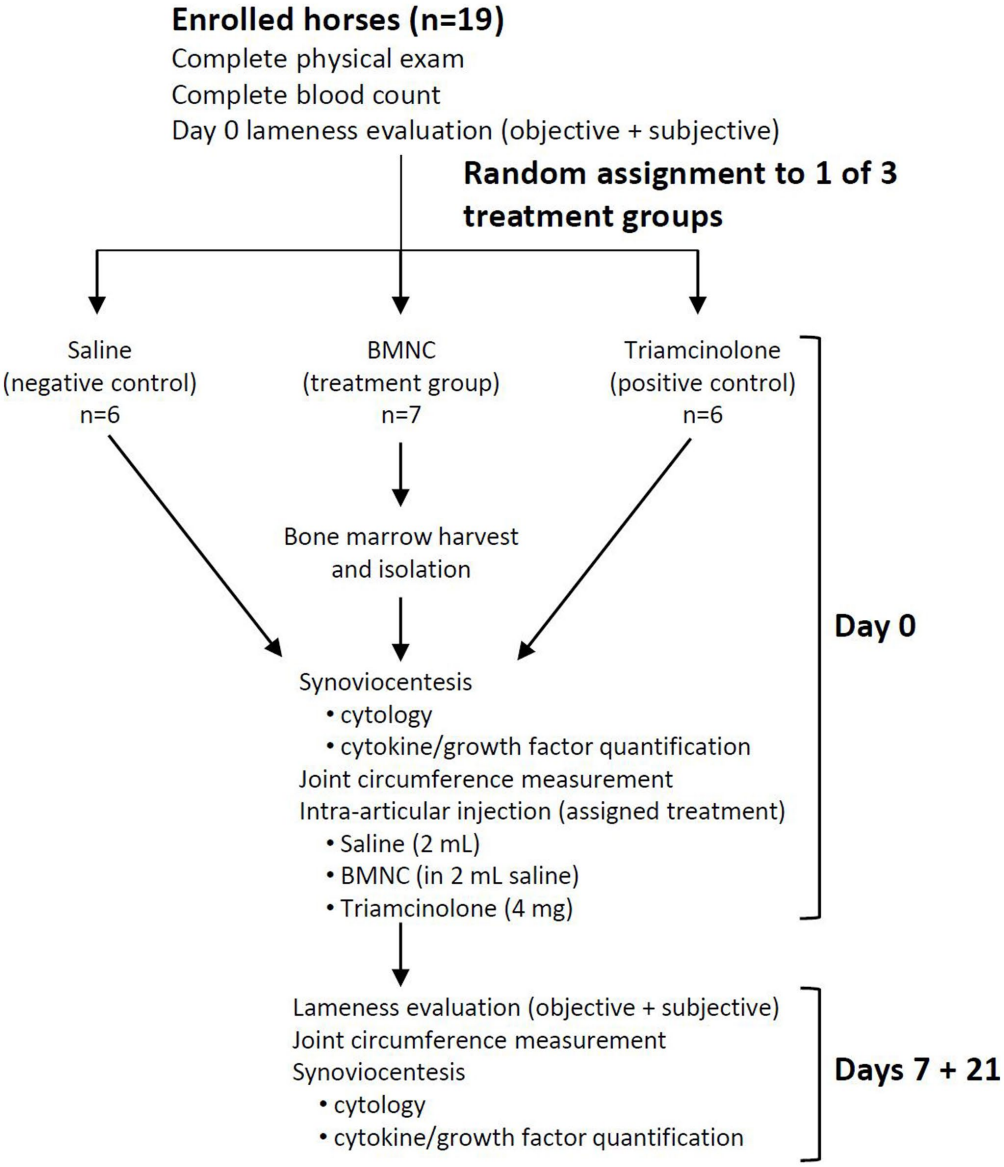
Schematic outlining study design.

Following written client consent, horses were assigned to 1 of 3 treatment groups using a random number generator (Excel, Microsoft Office 2019): saline (negative control, *n* = 6), triamcinolone (positive control, *n* = 6), or BMNC (treatment group, *n* = 7). A 7-day washout period was observed following intra-articular anesthesia. Horses underwent repeat lameness scoring to establish baseline lameness for the study on day 0. Horses were re-evaluated at 7 and 21 days following treatment to assess lameness, collect synovial fluid for cytology and cytokine/growth factor analysis, and to perform joint circumference measurements (Figure 1). The 7 and 21 day follow-up was selected to correlate with the time points from previous studies investigating the effects of BMNC *in vivo* in horses (41, 45).

Exclusion criteria included intra-articular injection of medication within 3 months prior to screening, severe co-morbidity (including complete intra-articular fractures and concurrent ligamentous and tendinous injuries), and administration of other systemic medications aimed at treating the musculoskeletal system (i.e., non-steroidal anti-inflammatory drugs, polysulfated glycosaminoglycans and nutraceuticals) within 4 weeks of study enrollment or during the 3 weeks of the study itself. Horses with lameness localized to the proximal or distal interphalangeal joints or distal tarsal joints were excluded due to the decreased likelihood of obtaining adequate volumes of synovial fluid.

### Objective and subjective lameness evaluation

Objective lameness evaluations were performed with a body-mounted inertial sensor system (Lameness Locator^®^) while horses were trotting in a straight line on a hard or firm surface. Sensors were attached to each horse as previously described (53–55). Briefly, each horse was instrumented with a poll (head), right forelimb pastern, and pelvis sensor device secured in place for the examination. Horses were lightly sedated using xylazine (0.3 mg/kg IV) to ensure steady, consistent inertial sensor readings (56). Forelimb lameness was considered present when the vector sum (*VS*) of MaxHDiff and MinHDiff >8.5 mm (53–55). If the *VS* was a positive value (MinHDiff is >0), lameness was considered localized to the right forelimb. A negative *VS* (MinHDiff is <0) indicated a left forelimb lameness. Data collected from vertical pelvic movement were evaluated using mean DIFFMAX and DIFFMIN. At the time horses were trotted for objective lameness assessment, the evaluation was video recorded for later subjective, blinded grading by 5 experienced clinicians (SHB, CRB, LAD, RSP, BCM) using the same published grading scale used for study enrollment (0–5) (51).

### Bone marrow harvest

Bone marrow aspirates for horses in the BMNC-treated group were performed immediately prior to intra-articular BMNC injection, as previously described (41, 57, 58). Horses were sedated with detomidine hydrochloride (0.01 mg/kg IV) and butorphanol tartrate (0.01 mg/kg IV). To minimize bias, the sternum of all horses, regardless of treatment, was clipped, aseptically prepared, and the skin, subcutaneous tissue and underlying musculature anesthetized with 2% lidocaine hydrochloride (0.5–2 mg/kg). Sham bone marrow aspirates were not performed on horses in the saline- or triamcinolone-treated groups, as lack of aspirate was not deemed to influence the outcome and was considered an invasive and therefore unnecessary procedure for client-owned horses. Bone marrow aspirates were collected from the 4th and 5th sternebrae (25 mL each site) using an 8-gauge Komiyashiki needle or 11-gauge Jamshidi needle and a heparinized 60 mL syringe (15,000 IU/aspirate) (18, 41, 57, 58).

### Bone marrow mononuclear cell isolation

In a laminar flow hood or portable clean bench, bone marrow aspirate was filtered (200 μm blood administration set), gently layered over 2.5 mL of Ficoll-Paque^™^ Plus (GE Healthcare Life Sciences) in sterile 15 mL conical tubes, and centrifuged (500 x g for 30 min at 4°C) (41, 57). Supernatant plasma was aspirated and discarded to an identifying mark 1.5 mL above the Ficoll ring. Mononuclear cells on Ficoll-Paque^™^ were aspirated, transferred to a sterile 50 mL conical tube, washed twice in phosphate buffered saline, centrifuged (400 x g for 10 min at 4°C), counted using a hematocytometer, and resuspended in phosphate buffered saline (20 × 10^6^ cells/mL). BMNC viability was confirmed by trypan blue dye exclusion (Sigma-Aldrich) (59). Isolated BMNC were maintained in sterile conical centrifuge tubes at 4°C to preserve viability and prevent clumping during transport from the laboratory to the clinic (41, 57). Cells in excess of those required for injection were cryopreserved for future studies.

### Synoviocentesis, cytology, and joint treatment

Affected joints were prepared aseptically for synoviocentesis and synovial fluid (~2 mL) was aspirated and aliquoted in EDTA for cytology (Virginia Tech Animal Laboratory Services, Blacksburg, VA) and in Protein LoBind^®^ tubes (Eppendorf^®^, Westbury, CT) for cytokine and growth factor quantification. Synovial fluid samples for cytology were analyzed using an automated processor (ADVIA 2120 hematology 173 Analyzer, Siemens Healthcare Diagnostics, Inc., Tarrytown, NY). Differential cell counts were performed by a veterinary clinical pathologist (SB). EDTA-free samples for cytokine and growth factor quantification were centrifuged (12,000 x g for 10 min at 4°C) and the supernatant stored at −80°C in Protein LoBind tubes. After synovial fluid was aseptically obtained for cytology and cytokine/growth factor quantification, and in the same synoviocentesis procedure, all joints received the assigned treatment in a total volume of 2 mL (saline, 20 × 10^6^ BMNC, or 4 mg triamcinolone).

### Joint circumference measurement

Joint circumference was used as a means of assessing soft tissue swelling and synovial effusion. The location of measurement was marked by clipping the hair for repeat measurements in the same location. Joint circumference (cm) was measured twice using a flexible measuring tape and averaged. All measurements were performed by a single observer (JBE) at days 0, 7 and 21 prior to synoviocentesis. To account for the possible differences in relative response between different joints, joint circumference on days 7 and 21 were normalized to the baseline value on day 0, resulting in a percentage change in circumference.

### Follow-up evaluation

Following treatment, horses were monitored for 24 h for attitude, appetite, temperature, pulse, respiratory rate, signs of local inflammation, measurement of joint circumference, and lameness in the stall. Evaluation outside the stall was performed at 24 h, at the walk only to minimize the possibility of joint trauma immediately following injection. Horses were discharged after evaluation at 24 h with instructions for 7 days stall rest, followed by 7 days stall rest with 15 min hand walking twice a day, and finally 20 min of trot under saddle for the final 7 days before re-evaluation. Horses were re-evaluated 7 and 21 days following treatment by performing a complete physical examination, measurement of joint circumference, and lameness evaluation by subjective and objective (Lameness Locator^®^) methods. Examinations were video recorded for later scoring by 5 blinded observers (SHB, CRB, LAD, RSP, BCM) and synovial fluid was collected under sedation from the treated joint for repeat cytology and cytokine/growth factor quantification.

### Cytokine and growth factor quantification

Aliquots of synovial fluid were thawed and digested with 10 mL hyaluronidase solution (100 IU testicular hyaluronidase in acetate buffer; LS005474; Worthington Biochemical, Lakewood, NJ, United States) in 200 mL of synovial fluid and incubated for 30 min at 37°C. Samples were then centrifuged for 10 min at 12,000 x g and 4°C to remove any particulate matter and the supernatant was recovered (Protein LoBind microfuge tubes) (44). Growth factors and anti- and pro-inflammatory cytokines and chemokines (FGF-2, GM-CSF, IL-1β, IL-6, MCP-1, IL-10, TNF-α, SDF-1(a + b), IGF-1, IL-1ra) were quantified by equine multiplex bead-based assay (Eqcttmag-93 K, Milliplex Map Equine chemokine/cytokine, Luminex 200 plate reader; MilliporeSigma). Manufacturer’s modifications to include IGF-1, SDF-1, and IL-1ra and to validate inclusion of one additional point at the lower end of the standard curve to maximize detection of low analyte concentrations were included. A full panel of 23 available cytokines and growth factors were screened using normal, OA and inflamed equine synovial fluid and those that were undetectable across these samples were eliminated from the multiplex. The selected cytokines and growth factors included in our study were based on prior publications and a knowledge of pathophysiology related to OA and BMNC (18, 41, 44, 60). Prostaglandin E_2_ was quantified by ELISA as previously described (R&D Systems, Minneapolis, MN, United States; SpectraMax M5 plate reader; Molecular Devices, San Jose, CA, United States) (44). Based on our previous experience with equine synovial fluid samples, a dilution of 1:2 was selected for PGE_2_ quantification, and no dilution was deemed necessary for the Milliplex assay (44).

### Statistical analysis

Preliminary data from a diverse study population such as the one we recruited were not available for sample size calculation. A sample size of 6 horses per group was therefore estimated heuristically based on previous studies (2, 37, 41, 45) and using an estimated effect size of 20% and standard deviation of 12 for the objective lameness data (vector sum), with *α* = 0.05 and *β* = 0.80. Normal probability plots were inspected to assess distribution properties of the data. Joint circumference and objective lameness data were log transformed to stabilize the model. All data are expressed as median and range. A Kappa statistic was used to quantify inter-observer variation for subjective video analysis of lameness. For subjective lameness evaluation, Friedman’s test was used to compare days within each treatment and the Kruskal-Wallis test was used to compare treatments at each time point. Lameness Locator^®^ data were analyzed for 17 horses with forelimb lameness. Two horses with pelvic limb lameness were excluded because of the inability to handle pelvic limb data in an identical manner as forelimb data. Lameness Locator^®^ data were collated in Microsoft Excel and inertial lameness measurements were reduced to 0 to represent a sound horse or one that then switched to lameness on the opposite limb. The Diff Max (push off lameness) or Diff Min (impact lameness) measurements were normalized to positive measurements, and entered under the limb that was lame (treated or control). Data were normalized to the baseline value on day 0 to create a percentage of baseline lameness and joint circumference on days 7 and 21. Data analysis for Lameness Locator^®^ and joint circumference data was performed using General Estimating Equations (GEE) to assess the effects of treatment and time. The interaction between treatment and time was further analyzed to compare treatments at each time point and to compare time points within each treatment. Where appropriate, *p* values were adjusted for multiple comparisons using the Tukey–Kramer test. All analyzes were performed using commercial software (SAS version 9.4, SAS Institute, Inc., Cary, NC). Significance was set at *p* < 0.05.

## Results

### Study population, BMNC isolation, and intra-articular injections

Nineteen adult horses (11 castrated males and 8 females) 3–16 years of age met the inclusion criteria for the study, including 18 Thoroughbreds and 1 American Quarter Horse. Of the 19 horses included, a single limb lameness was isolated to the forelimb in 17/19 (89.5%) and the hindlimb in 2/19 horses (10.5%; Table 2). BMNC were successfully isolated from all 7 horses in the treatment group with no adverse effects noted at the harvest site or following intra-articular injection in any horses. BMNC numbers varied by horse and age; however, the number isolated from each horse was well in excess of what was needed for treatment. Cell viability following isolation was 80–90%. Synoviocentesis and intra-articular injections were successfully performed in all horses. BMNC injections was performed within 4 h of bone marrow harvest in all horses and the time from bone marrow harvest to BMNC isolation decreased as experience with the isolation protocol and cell counting increased. All but one horse went on to be adopted and used for light trail riding to jumping (Table 2). One horse in the saline-treated group remained lame based on owner report. Detailed follow-up was not part of our study design.

**Table 2:** Signalment, affected joint, treatment, and outcome for horses enrolled in study.

Horse #	Age (years)	Breed	Sex	Affected Joint	Treatment	Outcome
1	3	TB	Gelding	R radiocarpal	BMNC	Adopted for NCFR
2	7	TB	Gelding	L middle carpal	BMNC	Adopted for light trail riding
3	3	TB	Mare	R radiocarpal	Triamcinolone	Adopted for NCFR
4	3	TB	Gelding	R radiocarpal	Saline	Adopted for NCFR
5	3	TB	Mare	L middle carpal	BMNC	Adopted for NCFR
6	4	TB	Mare	R middle carpal	Saline	Adopted for NCFR
7	6	TB	Gelding	RF MCP	Saline	Remained lame
8	6	TB	Gelding	RF MCP	BMNC	Adopted NCFR
9	3	QH	Gelding	R tibiotarsal	Triamcinolone	Returned to previous use
10	10	TB	Gelding	LF MCP	BMNC	Increased level of use; cantering + low level jumping
11	6	TB	Mare	LF MCP	Triamcinolone	Returned to previous use
12	7	TB	Gelding	R middle carpal	Saline	Adopted NCFR
13	11	TB	Mare	LF MCP	Triamcinolone	Adopted NCFR
14	5	TB	Mare	RH MCP	BMNC	Switched to RF lameness; used for low level jumping
15	16	TB	Gelding	RF MCP	Triamcinolone	Adopted NCFR
16	3	TB	Mare	R middle carpal	Triamcinolone	Adopted NCFR
17	3	TB	Mare	L middle carpal	Saline	Adopted NCFR
18	6	TB	Gelding	RF MCP	BMNC	Adopted NCFR
19	3	TB	Gelding	L middle carpal	Saline	Adopted NCFR

TB, Thoroughbred; QH, Quarter Horse; BMNC, bone marrow mononuclear cells; R, right; L, left; RF, right front; LF, left front; MCP, metacarpophalangeal; NCFR, new career following racing.

### Clinical exam and synovial fluid cytology

**Figure 2:**
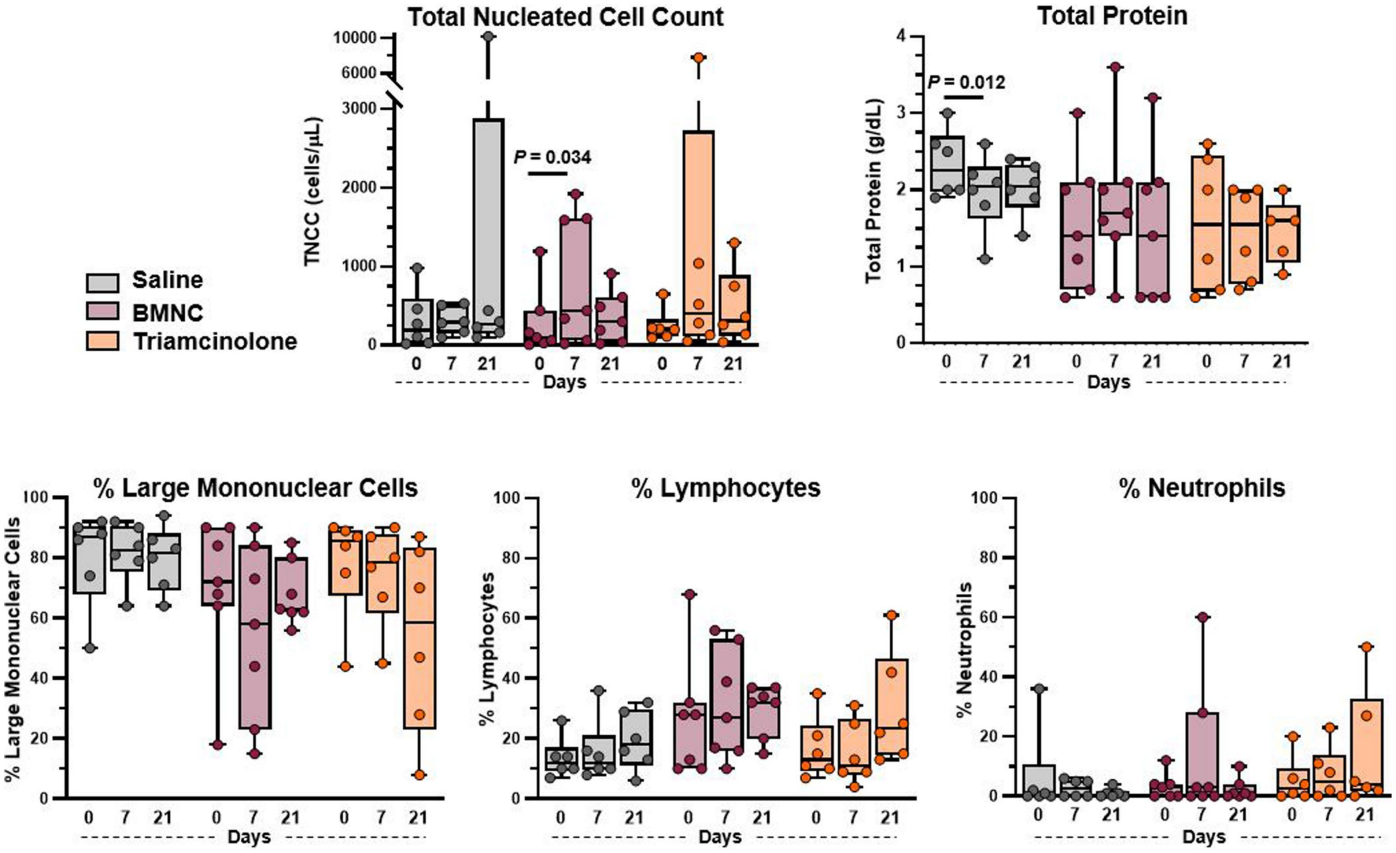
Synovial fluid cytology from saline-, BMNC-, and triamcinolone-treated joints over time. Horizontal bars above box plots indicate significant differences. Median TNCC remained <500 cells/uL and median TP was <2.25 g/dL for all treatments at all time points. Boxes represent interquartile range with median and whiskers represent range. Individual horses are shown as colored dots.

Body temperature, heart rate, respiratory rate, and appetite remained normal for all horses throughout the study. Horse 9 had increased synovial effusion on day 7 following triamcinolone injection, which resolved by day 21. Horse 17 in the saline group showed signs of transient post-injection synovitis with increased lameness between days 1 and 4 that was resolved by day 7 without intervention. Additionally, Horse 11 in the triamcinolone group had increased lameness at day 21 compared to baseline. Median TNCC remained ≤440 cells/uL at all time points for all treatment groups (Figure 2). Median TNCC at day 7 in the BMNC group (440 cells/uL) was significantly higher than at day 0 (100 cells/uL; *p* = 0.034). Mononuclear cells were the predominant cell type across treatments at all time points. Median TP decreased significantly between 0 and 7 days in the saline-treated group (Figure 2; *p* = 0.012). There were no other differences detected between groups or over time for TNCC, TP, or differential cell counts.

### Joint circumference

**Figure 3:**
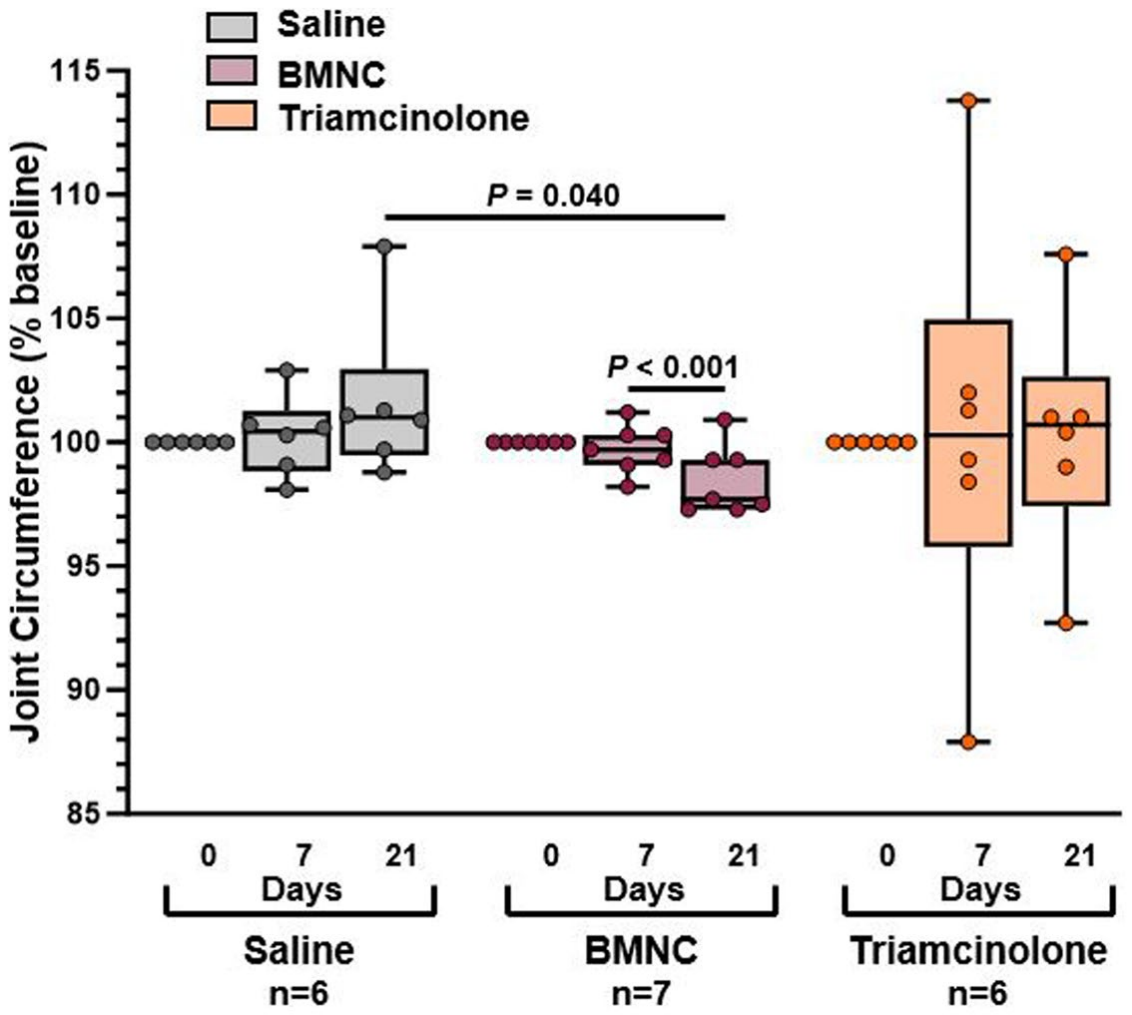
Joint circumference reported as percentage of baseline measurements for saline-, BMNC-, and triamcinolone-treated horses over time. Horizontal bars above box plots indicate significant differences. Boxes represent interquartile range with median and whiskers represent range. Individual horses are shown as colored dots.

Overall, when considering all horses regardless of treatment, joint circumference did not differ by treatment group (*p* = 0.124) or over time (Figure 3; *p* = 0.973). However, within the BMNC-treated group, joint circumference did decrease significantly over time from day 7 to 21 (*p* < 0.001). Joint circumference, as % baseline, in the BMNC-treated group was significantly lower compared to the saline-treated group on day 21 (*p* = 0.040). There were no significant differences in joint circumference over time for the saline (*p* = 0.126) and triamcinolone groups (*p* = 0.949).

### Lameness evaluation

**Figure 4:**
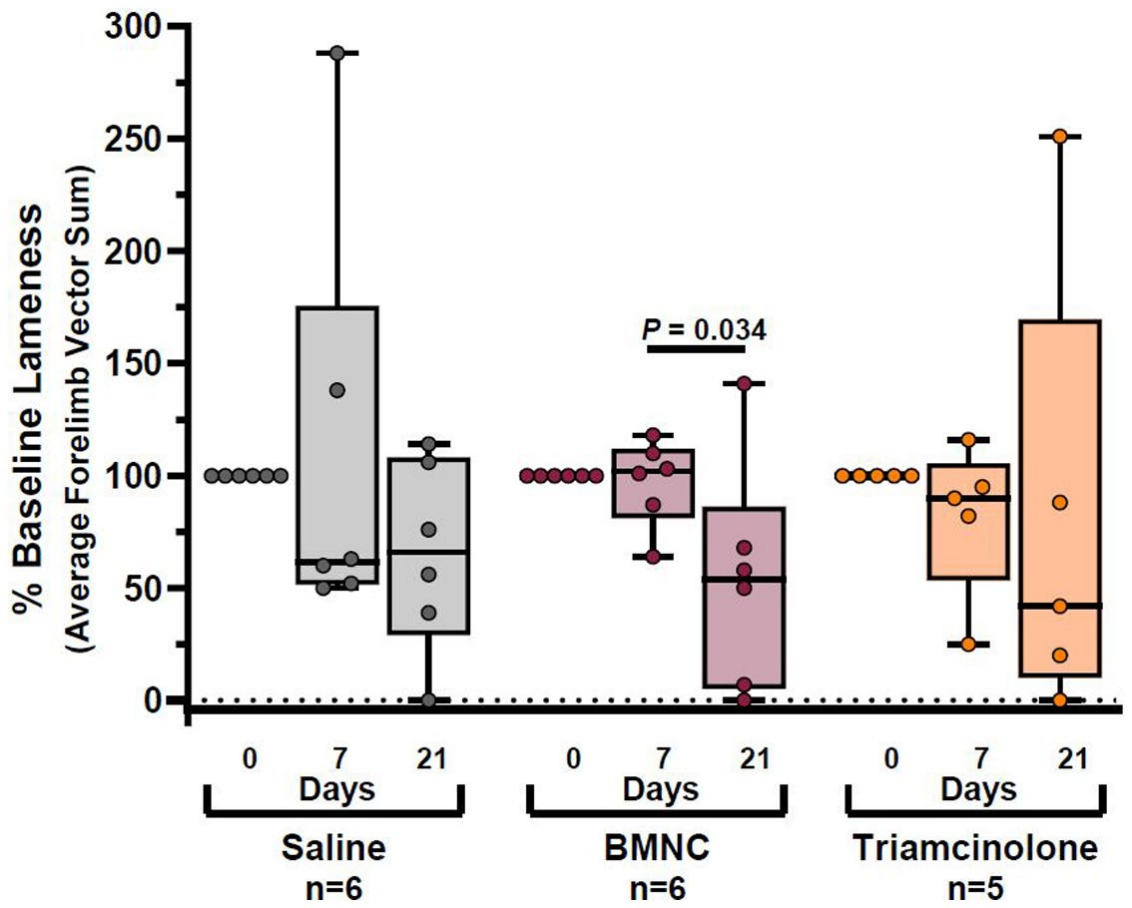
Objective lameness scores reported as percentage of baseline lameness for saline-, BMNC-, and triamcinolone-treated horses over time. Horizontal bars above box plots indicate significant differences. Boxes represent interquartile range with median and whiskers represent range. Individual horses are shown as colored dots.

Objective lameness data for the 17 horses with forelimb lameness were included for statistical analysis. Two horses with hindlimb lameness (1 horse from each of the saline- and triamcinolone-treated groups) were excluded from objective lameness evaluation due to the inability to analyze hindlimb lameness using the same vector sums as the forelimbs. Overall, objective lameness, expressed as compared to baseline lameness (% baseline) did not differ by treatment group (*p* = 0.955); however, it did differ significantly over time (Figure 4; *p* = 0.004). Lameness decreased over time, but not significantly, in the saline- (*p* = 0.152) and triamcinolone-treated groups (*p* = 0.143). Lameness decreased significantly from day 7 to 21 in the BMNC- (*p* = 0.034).

**Figure 5:**
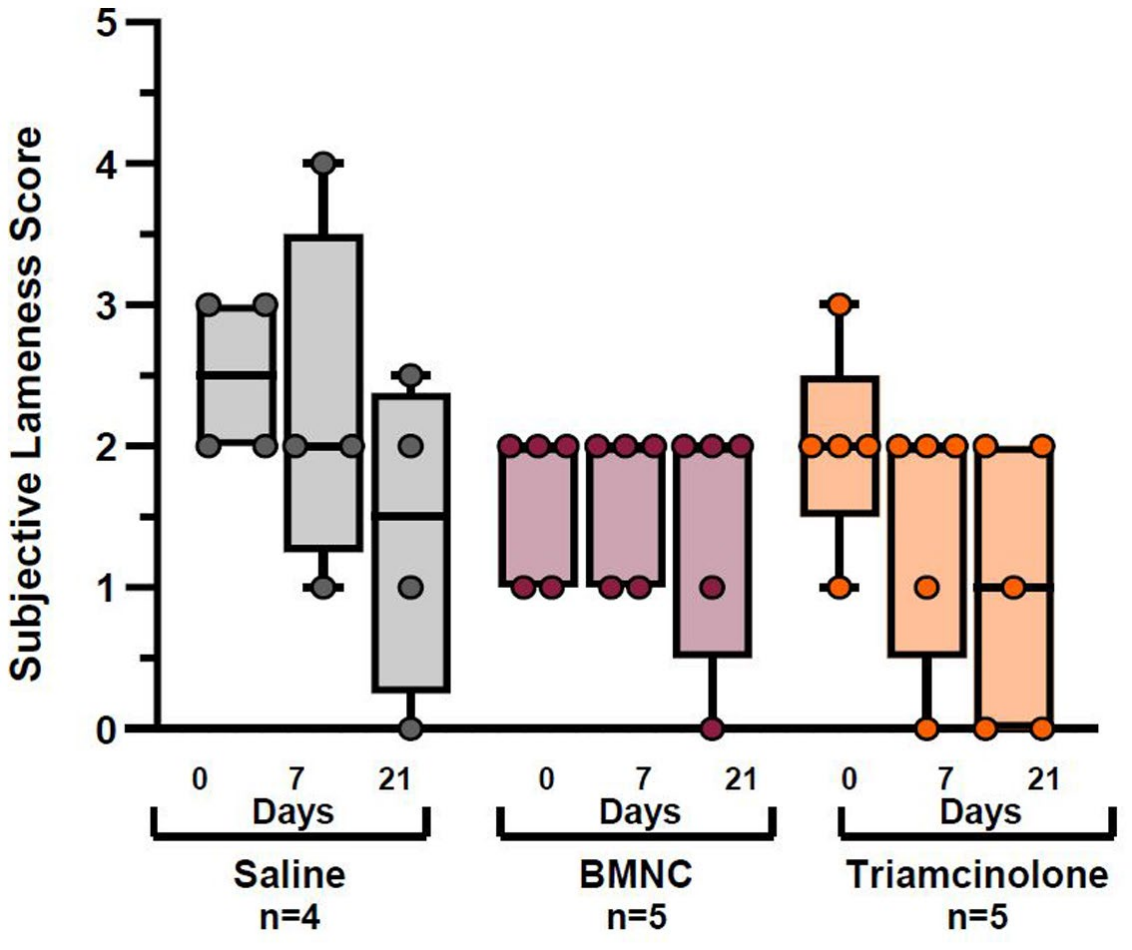
Subjective lameness scores for saline-, BMNC-, and triamcinolone-treated horses over time. Boxes represent interquartile range with median and whiskers represent range. Individual horses are shown as colored dots.

The same horses with hindlimb lameness excluded for analysis of objective lameness data were also excluded from subjective lameness analysis to maintain equivalent data sets for lameness evaluations. In addition, two horses from the saline group (# 7 and 19) and one horse from the BMNC group (# 8) were excluded from the subjective lameness data analysis because agreement among observers was so poor that no discernible pattern was evident for a logical analysis. As a result, 5 horses were included in the BMNC and triamcinolone groups and 4 horses in the saline group for subjective lameness analysis. There were no significant differences in subjective lameness evaluation scores between treatments at any time point or within any treatments over time (Figure 5). Interrater reliability was significantly different from 0 (Kappa = 0.204; 95% CI (0.146, 0.262); *p* < 0.001), indicating there was some, albeit poor, agreement between observers.

### Cytokine and growth factor quantification

**Figure 6:**
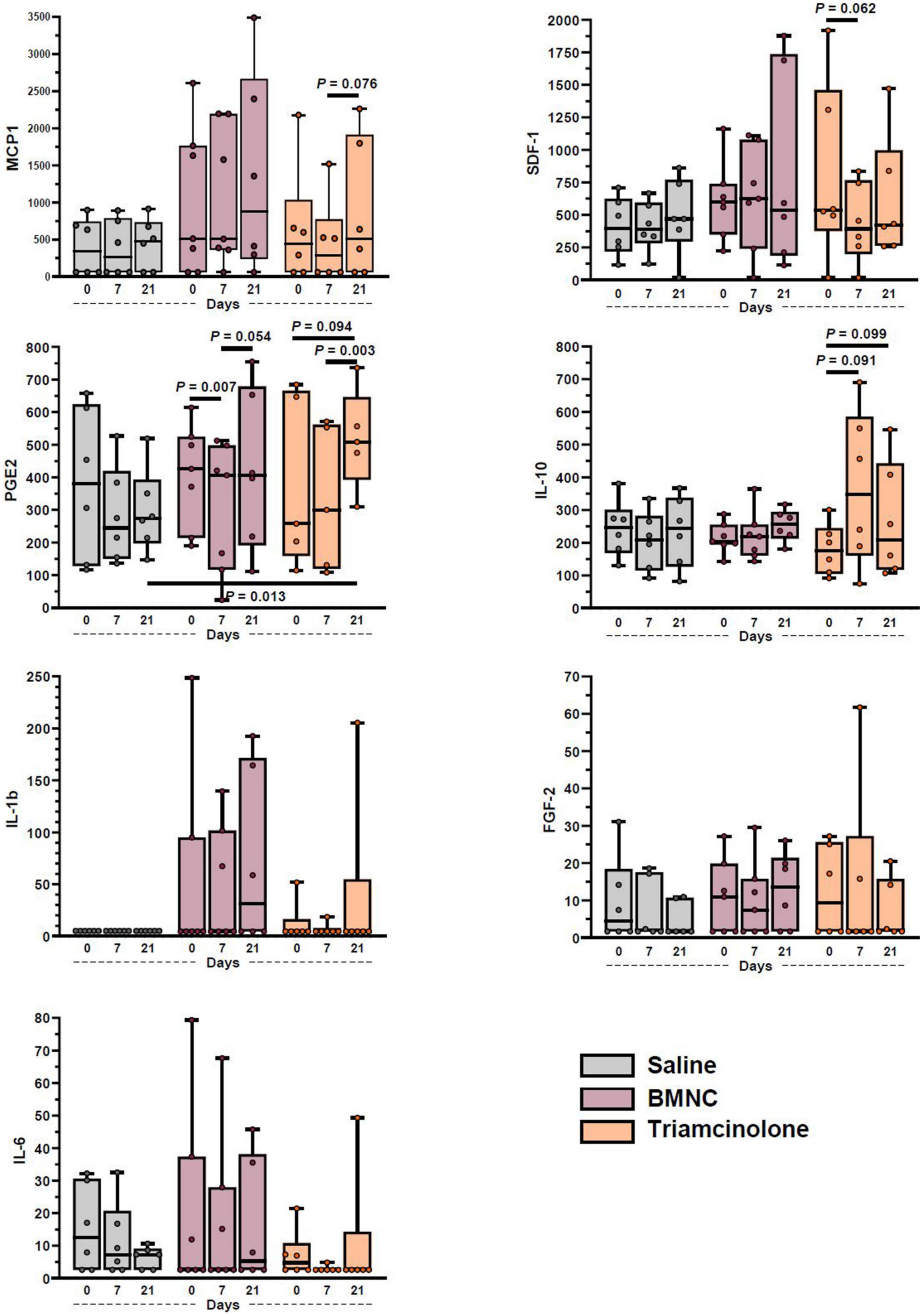
Synovial fluid cytokine and growth factor concentrations from saline-, BMNC-, and triamcinolone-treated joints over time. Horizontal bars above box plots indicate select pairwise differences between groups. Boxes represent interquartile range with median and whiskers represent range. Individual horses are shown as colored dots.

Concentrations of pro- (IL-1β, IL-6, GM-CSF, TNF-α, PGE_2_) and anti-inflammatory cytokines (IL-10, IL-1ra), chemokines (MCP-1, SDF-1) and growth factors (IGF-1, FGF-2) were quantified in synovial fluid at baseline, prior to treatment, and at 7 and 21 days following treatment (Figure 6). The following analytes were below detectable limits (listed in parentheses): GM-CSF (3.54 pg./mL; all samples); IL-1ra (3.98 pg./mL; all samples); IGF-1 (4.42 pg./mL; all samples except 1 horse at all time points); and TNF-α (0.88 pg./mL; all samples except 2 samples in 2 different horses). Detection of MCP-1, SDF-1a + b, IL-10, IL-6, IL-1β, FGF-2, and PGE_2_, was possible in the majority of samples; however, statistical analysis was only possible for MCP-1, SDF-1a + b, IL-10, and PGE_2_ due to limited numbers of detectable samples for IL-6, FGF-2, and IL-1β. Overall, concentrations of PGE_2_ varied significantly by time (*p* < 0.001), but not treatment (*p* = 0.630); there was a significant day*treatment interaction (*p* = 0.008). On day 21, median PGE_2_ concentrations were significantly higher in triamcinolone-treated compared to saline-treated joints (*p* = 0.013). Median PGE_2_ concentrations in BMNC-treated joints varied significantly over time (*p* < 0.001) and were higher on day 7 compared to day 0 (*p* = 0.070) and day 21 compared to day 7 (*p* = 0.054). Median PGE_2_ concentrations in triamcinolone-treated joints varied significantly over time (*p* = 0.002) and were higher on day 21 compared to day 0 (*p* = 0.094) and day 7 (*p* = 0.003). Median concentrations of SDF-1 overall varied by time (*p* = 0.003), but not treatment (*p* = 0.288); there was a significant day*treatment interaction (*p* < 0.001). In triamcinolone-treated joints, median SDF-1 concentrations varied over time (*p* = 0.001) with a decrease on day 7 compared to day 0 (*p* = 0.062). Overall, median MCP-1 concentrations varied significantly over time (*p* = 0.022), but not treatment (*p* = 0.171). Median MCP-1 concentrations in the triamcinolone-treated joints also varied significantly by time (*p* = 0.041), with a decrease between 7 and 21 days (*p* = 0.076). Median IL-10 concentrations in the triamcinolone-treated group increased significantly over time (*p* = 0.046), but not treatment (*p* = 0.708). Median IL-10 concentrations in the triamcinolone-treated joints also varied significantly by time (*p* = 0.046), with decreases between 0 and 7 (*p* = 0.091) and 0 and 21 days (*p* = 0.099).

## Discussion

Intra-articular BMNC injection in horses with moderate, naturally-occurring OA was associated with no adverse effects. BMNC was the sole treatment that resulted in decreased objective lameness, which was complemented by a concurrent decrease in joint circumference. The majority of lameness improvement in BMNC-treated horses occurred between 7 and 21 days. There were no significant improvements in lameness or joint circumference in the saline- or triamcinolone-treated groups. Importantly, synovial fluid parameters remained within normal limits for the 21 days following BMNC injection, with a small significant increase in TNCC at day 7; however, TNCC remained within the physiological range. This is the first controlled clinical trial investigating autologous intra-articular BMNC injection for the treatment of horses with naturally-occurring OA and provides objective data supporting a larger clinical trial. BMNC are autologous, readily available without extensive processing, and have the potential for sustained clinical effect and return to joint homeostasis by driving inflammation resolution.

Decreased lameness and inflammation observed following BMNC treatment in our study is consistent with published reports in clinical trials in people with knee OA (42, 43), and an experimental model of synovitis in horses (41). The transient increase in TNCC in the BMNC-treated group in our study remained within the physiological range, and may have been a direct response to the nucleated cells injected into the joint and/or to a recruitment of endogenous nucleated cells, possibly to initiate a pro-resolving response.

As hypothesized, BMNC treatment significantly reduced objectively-assessed lameness and were consistent across all horses in the group. Worsening of lameness, as observed for one horse in the triamcinolone-treated group, is a common finding in clinical practice both in horses and people, due to the short-lived effects of intra-articular corticosteroids, especially when tissue damage is extensive (61). The results from BMNC treatment in this study are consistent with human studies evaluating the use of intra-articular BMNC for knee OA (42, 43). A single dose of BMNC lasted more than 12 months, was considered superior to 3 weekly injections of hyaluronan, and demonstrated clinical improvement in 96% of BMNC-treated patients (42, 43). Intra-articular injection of enriched peripheral blood-derived mononuclear cells, an alternate source of anti-inflammatory macrophages, showed significant improvements in knee pain and Knee injury and Osteoarthritis Outcome Score for up to 24 months in a recent clinical trial in people (62). BMNC have the advantage of providing a much higher proportion of macrophage progenitors (40–70%) compared to peripheral blood mononuclear cells (10–19%). The remaining mononuclear cells in peripheral blood mononuclear cells are lymphocytes. BMNC injection was the only treatment resulting in significantly reduced joint circumference, a measure of soft tissue swelling and synovial effusion, and therefore an indirect measure of inflammation (31, 63). Reduced gross inflammation is consistent with our experimental study investigating the intra-articular effects of BMNC in horses (41, 44). A single injection of BMNC was selected because of the expected lasting effects of BMNC and to align with previous human and equine studies (41–43, 45). The potential for lasting improvement following treatment with mononuclear cells is an exciting advancement in the treatment of osteoarthritis and warrants further long-term clinical trials.

Cytokines, growth factors and chemokines are known to play key roles in inflammation, inflammation resolution and tissue repair and can be produced by both macrophages and fibroblasts in the synovial lining (64). Statistical analysis was possible on only four cytokines (MCP-1, SDF-1a + b, IL-10, and PGE_2_), with a further three cytokines detectable in the majority of samples (IL-6, IL-1β, and FGF-2). These findings are consistent with our previous equine studies (41, 44, 60). Our current results are overall consistent with our previous results; however, direct comparisons are not possible based on different study designs. IL-1β and TNF-α were essentially undetectable in these clinically affected joints, similar to previous reports (60, 65, 66) and lending further support to the premise that the roles of IL-1β and TNF-α may not be as prominent as classically reported. In fact, current studies show that a short peak of IL-1β and TNF-α production, as observed in acute inflammation, is required to prime macrophages and infiltrating monocytes towards a regulatory response (40, 44, 67). The role of PGE_2_ in OA is complex. It is involved in inflammation as well as being involved in anti-inflammatory and anabolic roles, chondrocyte protection and tissue repair (25, 68, 69). Because of its complex roles, PGE_2_ concentrations likely vary depending on the response to injury and may not be an ideal marker for joint inflammation. The increased median PGE_2_ concentration in triamcinolone-treated joints was unexpected. While a large cohort of horses in each group would be required for strengthening the power of this finding, it warrants further mechanistic investigation. With a relatively small sample size, horse-to-horse variations in disease stage and response to disease, the limited time points for sample collection in our study, and potentially very small changes in growth factor, cytokine and chemokine levels, it is possible that clinically relevant changes, as observed in our previous study (41), went undetected.

In equine and murine chronic inflammatory airway disease, BMNC exhibited potent anti-inflammatory effects comparable to corticosteroids (32, 45). Intratracheal BMNC delivery in horses affected with recurrent airway obstruction resulted in 30-fold higher IL-10 concentrations in bronchoalveolar lavage fluid compared to horses treated with dexamethasone (45). In a murine model of airway disease, BMNC depleted of macrophages eliminated the reparative and remodeling processes initiated by BMNC therapy, providing further evidence of the potent anti-inflammatory potential of BMNC-derived macrophages (32). Our previous *in vivo* study of BMNC used to treat experimental synovitis showed that increases in the number of IL-10-producing cells occurred within the first 96 h following BMNC injection (41). Therefore, comparable increase in IL-10 may have been missed based on the timeline of this study. While corticosteroids are known to induce increased IL-10 expression in macrophages (70, 71), the concurrent inhibition of other important homeostatic pathways prevents beneficial effects on cartilage preservation (70). While reduced lameness and soft tissue inflammation noted in our horses could be related to early increased concentrations of IL-10, other specialized pro-resolving mediators not measured in our study are likely to have contributed to such an effect (72, 73).

While changes in inflammatory mediators in the joint occur as early as 96 h following treatment with BMNC, marked clinical improvement was observed in BMNC-treated horses between 7 and 21 days, similar to the timing for horses with airway disease treated by BMNC by intratracheal infusion. These findings suggest that assessment of the therapeutic effects of BMNC should include time points at least 2 weeks after treatment, as opposed to only 7 days as for corticosteroid injections. Although experimentally inflamed joints recover normal histology 6 days following BMNC treatment, recent reports show that the effect of BMNC-mediated resolution of inflammatory pain in sensory neurons may take longer, peaking 8 to 9 days after treatment (74).

Potential limitations of our study include a relatively small sample size and documented challenges in detecting subtle differences in lameness in horses. Even with our small sample size, we were able to detect differences in our BMNC treatment over time and between BMNC and saline-treated horses for joint circumference. Subjective scoring of video archives can be inconsistent between observers, as demonstrated in ours and multiple other studies (53, 75, 76), and can vary within the same evaluation period. A study evaluating the repeatability of subjective evaluation found that when the mean AAEP lameness score was ≤1, graders agreed only 61.9% of the time (53). Moreover, clinicians only agreed 51.6% of times when asked to choose if the horse was lame and what limb was affected (53). Despite efforts to consistently position the video camera, video quality varied and was affected by factors such as sunshine, shadows, and handler technique, which were uncontrolled variables in this multicenter study. Change over time can be measured quantitatively with the Lameness Locator^®^ and has been shown to be more sensitive in the detection and diagnosis of lameness compared to subjective evaluation (76). Horses with lameness localized to different joints were included in our study to include a diverse manifestation of disease and to enable timely case enrollment. Lack of selection of a single specific joint and/or lack of equal distribution of joints between treatments has the potential to confound the effects of our treatments, especially when considering the effect on joint circumference. Changes in joint circumference were normalized to the baseline value to account, in part, for these potential differences between joints.

Additional details of our study design warrant discussion, including dosage of BMNC selected and use of saline as the negative control. Though cell dosage as it correlates to clinical effects is a relevant topic of discussion, data are unavailable at present for making a evidence-based decision (42). Our dose was selected based on cell numbers used for MSC therapy, our *in vitro* work with BMNC and a somewhat educated guess. We extrapolated from the optimal 2 ×10^6^ cells per well in 1 mL of culture medium, using a estimated 10 mL of synovial fluid per joint to reach our 20 ×10^6^ BMNC/joint. One human study used 45.56 ± 34.94 × 10^6 per joint and stated in their discussion that they observed no correlation between cell dose and clinical effect despite subjective observations to the contrary (43). Dose effect would certainly be an important variable to consider for future studies. Saline served as the negative control for our study because it was the vehicle in which the BMNC were delivered and therefore provided the most relevant comparison. Recent work has called into question the use of a saline placebo in human OA studies based on findings that saline may exert active analgesic effects (77) and result in clinical improvement after injection (78). These findings suggest that evaluation of studies using saline injection as a placebo control could be confounded. Such as response to saline injection has not been reported in horses and whether the same effect occurs in horses is an interesting question, but is unknown at present. An alternative to saline injection in our study, such as a simple arthrocentesis without injection, could have been considered. However, saline provided the most direct comparison for our study such that the only difference between the saline-treated control group and the BMNC-treated group was the presence of BMNC. Results from a needle puncture alone would have been difficult or impossible to interpret because of the presence of two factors (saline and BMNC) compared to controls. In addition, we know from our *in vivo* experimental model of synovitis (41) that saline-treated joints remained grossly and histologically abnormal at 6 days following injection, whereas BMNC-treated joints were comparable to healthy joints. Reduced exercise alone may have played as large or larger a factor in the response of saline-treated controls over time. Our saline-treated horses did not show significant improvements in lameness, as described in people (77, 78).

Our work focused only on BMNC. Recent work suggests a strong relationship between tissue-resident fibroblasts and macrophages (64, 79). Future investigations focused on how BMNC may alter fibroblast gene expression would be interesting and could help uncover key interactions important in targeting return to homeostasis. BMNC injection may effect quantitative changes to the proportions of cells that makeup key cellular checkpoints important in suppressing inflammation and restoring joint homeostasis (64). A key topic that would be interesting to incorporate in future studies is how macrophage phenotypes change within OA joints before and after BMNC injection. Theoretically, the relatively naïve macrophage progenitors (BMNC) respond to the abnormal synovial environment in OA by developing a dynamic range of hybrid phenotypes and by providing necessary physiologic cues to trigger tissue repair by tilting the scales toward a homeostatic response (18). Unfortunately, macrophage polarization *in vivo* is much more complicated than was originally described by early *in vitro* studies using supraphysiological concentrations of lipopolysaccharide and cytokines in defined culture media (18). At present, our knowledge of targets to study macrophage phenotype *in vivo* and available reagents for equine-specific surface markers are insufficient to accurately design studies to inclusively evaluate the multifaceted immune response, including inflammation and its resolution. Hopefully as the field advances, these studies will become possible.

The results of our clinical trial investigating the use of intra-articular BMNC for naturally-occurring OA demonstrated significant improvement in objective lameness and soft tissue swelling in BMNC-treated horses and warrant a larger clinical trial with long-term follow-up. The data obtained in this study may be used to calculate an appropriate sample size for a larger clinical trial based on the final design of such a study. Minimal processing was required to produce autologous, point-of-care BMNC, which caused no adverse effects when injected intra-articularly. Overall, our results agree with findings from an experimental model of synovial inflammation in horses, as well as clinical trials of intra-articular BMNC for OA-affected human patients (41–43). Findings from *in vitro* studies suggest that clinical improvements from BMNC are associated with an early pro-inflammatory response that triggers sustained increased expression of known drivers of inflammation resolution, such as IL-10, IGF-1 and peroxisome proliferator-activated receptor-gamma, and isoprenoid biosynthesis (40). However, further *in vivo* studies are required to explore these and other mechanisms by which BMNC drive synovial inflammation resolution. Our clinical findings support further investigations into these specific mechanisms by which BMNC may lead to long-lasting resolution of joint inflammation and the promotion of tissue repair for the treatment of OA.

## Data availability statement

The original contributions presented in the study are included in the article/supplementary material, further inquiries can be directed to the corresponding author.

## Ethics statement

The animal studies were approved by Virginia Tech Institutional Animal Care and Use Committee. The studies were conducted in accordance with the local legislation and institutional requirements. Written informed consent was obtained from the owners for the participation of their animals in this study.

## Author contributions

JE: Conceptualization, Data curation, Formal analysis, Funding acquisition, Investigation, Methodology, Writing – original draft. BM: Conceptualization, Data curation, Formal analysis, Funding acquisition, Investigation, Methodology, Writing – original draft, Writing – review & editing. SaB: Investigation, Writing – review & editing, Methodology. SoB: Data curation, Investigation, Writing – review & editing. CB: Investigation, Methodology, Writing – review & editing. RP: Investigation, Methodology, Writing – review & editing. SW: Investigation, Methodology, Writing – review & editing, Data curation, Formal analysis. LD: Data curation, Formal analysis, Investigation, Methodology, Writing – review & editing, Conceptualization, Funding acquisition, Project administration, Supervision, Writing – original draft.

## Abbreviations

OA: osteoarthritis
BMNC: bone marrow mononuclear cells
IL: interleukin
IL-10: interleukin-10
IGF-1: insulin-like growth factor-1
PGE_2_: prostaglandin E_2_
SDF-1: stromal derived factor-1
IL-1ra: interleukin-1 receptor antagonist
TNCC: total nucleated cell count
TP: total protein
MCP-1: macrophage chemoattractant protein
GM-CSF: granulocyte-macrophage colony-stimulating factor
FGF-2: basic fibroblastic growth factor
